# Implementation of a Hepatitis C Screening Program for At-Risk Former Soviet-Bloc Immigrants in a Large Health Maintenance Organization

**DOI:** 10.1093/ofid/ofag278

**Published:** 2026-05-06

**Authors:** Daniella Rahamim-Cohen, Bar Cohen, Dor Atias, Afif Nakhleh, Ori Liran, Izana Kaplan Lavi, Joseph Azuri, Limor Adler, Shirley Shapiro Ben David

**Affiliations:** Division of Health, Maccabi Healthcare Services, Tel Aviv, Israel; Division of Health, Maccabi Healthcare Services, Tel Aviv, Israel; Division of Health, Maccabi Healthcare Services, Tel Aviv, Israel; Department of Epidemiology and Preventive Medicine, School of Public Health, Gray Faculty of Medical and Health Sciences, Tel Aviv University, Tel Aviv, Israel; Division of Health, Maccabi Healthcare Services, Tel Aviv, Israel; Institute of Endocrinology, Diabetes and Metabolism, Rambam Health Care Campus, Haifa, Israel; Azrieli Faculty of Medicine, Bar-Ilan University, Safed, Israel; Division of Health, Maccabi Healthcare Services, Tel Aviv, Israel; Family Medicine Department, Gray Faculty of Medical and Health Sciences, Tel Aviv University, Tel Aviv, Israel; Division of Health, Maccabi Healthcare Services, Tel Aviv, Israel; Division of Health, Maccabi Healthcare Services, Tel Aviv, Israel; Department of Epidemiology and Preventive Medicine, School of Public Health, Gray Faculty of Medical and Health Sciences, Tel Aviv University, Tel Aviv, Israel; Family Medicine Department, Gray Faculty of Medical and Health Sciences, Tel Aviv University, Tel Aviv, Israel; Division of Health, Maccabi Healthcare Services, Tel Aviv, Israel; Family Medicine Department, Gray Faculty of Medical and Health Sciences, Tel Aviv University, Tel Aviv, Israel; Division of Health, Maccabi Healthcare Services, Tel Aviv, Israel; Family Medicine Department, Gray Faculty of Medical and Health Sciences, Tel Aviv University, Tel Aviv, Israel

**Keywords:** HCV, screening, screening adherence

## Abstract

**Background:**

Hepatitis C virus (HCV) is a leading cause of liver disease worldwide. In 2021, Israel's Ministry of Health launched a national program to screen high-risk populations, especially immigrants from former Soviet Union countries, aiming to reduce HCV-related morbidity and mortality. This study evaluates the success of Maccabi Health Services (MHS) in implementing the screening program before and after the intervention, focusing on detection, treatment, and cure rates.

**Methods:**

A retrospective interrupted time-series cohort study was conducted among MHS members born in former Soviet Union countries, between 2019 and 2024. The intervention included proactive referrals for HCV antibody testing, educational messaging, and removal of bureaucratic barriers. Key outcomes were number of tests performed, positivity rates, polymerase chain reaction (PCR) confirmation, initiation of direct-acting antiviral treatment, and sustained virologic response (SVR).

**Results:**

Of 179 658 eligible members, 82.3% were screened after the intervention (compared to only 14.2% before). The rate of positive antibody tests dropped from 2.6% preintervention to 1%–1.1% postintervention. Ninety-three percent of antibody-positive individuals underwent PCR confirmation, with 52% testing positive. Eighty-four percent of PCR-positive patients started treatment, with higher initiation rates in the early intervention group. Sixty-nine percent of treated patients underwent SVR testing after treatment, with a median time of 106 days from treatment completion.

**Conclusions:**

The proactive intervention by MHS led to a significant increase in screening coverage, detection, and treatment among the at-risk population. Removing barriers, providing education, and regular reminders were key to the program's success. This structured approach can serve as a model for screening programs in other populations and countries.

Key points Actively approaching patients, sending reminders, and reducing bureaucratic and logistic hassles can support successful implementation of hepatitis C virus (HCV) screening programs. HCV screening programs can reach populations with different characteristics to patients tested due to clinical suspicion.

The hepatitis C virus (HCV) is one of the most common causes of liver disease globally. In 2024, the World Health Organization (WHO) estimated that 50 million individuals have chronic HCV infection, with 1 million new infections every year [1]. HCV is transmitted sexually, perinatally, or by exposure to blood [2, 3] and can lead to cirrhosis and hepatocellular carcinoma [4–6]. In 2014, direct-acting antiviral (DAA) agents were introduced, revolutionizing HCV therapy and replacing prior interferon drug regimens. Taken correctly, >95% of patients reach sustained viral response (SVR), thus curing most patients and significantly reducing morbidity and mortality [7–10].

These developments inspired hope to decrease the incidence of HCV, and in combination with efforts to prevent transmission, possibly start a process of eradicating the disease altogether. Due to the virus having no known nonhuman and latent cellular reservoirs, this is considered a feasible goal [11]. Therefore, the WHO and its member countries declared it their goal to reduce new HCV infections by 90% and HCV-related deaths by 65% by 2030 [12]. In response, many countries, including Israel, developed programs and strategies to achieve this goal [13]. DAAs were included in the Israeli Healthcare Basket as early as 2015 for patients with advanced liver fibrosis and HCV genotype 1. Eligibility criteria for DAAs were gradually expanded until 2018 [14], when all patients with positive HCV antibody testing were deemed eligible for treatment, regardless of genotype or fibrosis level under the universal coverage of the Israeli Healthcare Basket. During this time, a structured program was implemented to identify eligible patients for treatment, and an initial care cascade was formed within Maccabi Healthcare Services (MHS), the second-largest health maintenance organizations (HMOs) in Israel. In 2019 MHS initiated a much broader intervention program, focused on improving linkage to care for the population already diagnosed with positive HCV antibodies by improving patient-, provider-, and system-based barriers [15]. These programs were successful in shortening time to diagnosis and treatment and formed the basis for our 2021 screening program.

In 2021, the Israeli Ministry of Health announced a nationwide strategic effort to screen relevant populations for HCV actively [16] as the next step in HCV elimination. Previously, patients were sent for HCV testing if a clinical suspicion arose, such as jaundice, an elevation of liver enzymes, or prolonged fever. Furthermore, HCV testing was added as a screening test for pregnant women, within the battery of tests done in early pregnancy.

This program delegated the implementation of the screening process to the HMOs, which provide primary care services to the country's population. The program stipulates that HMOs actively approach high-risk patients and offer HCV antibody testing. If positive, a further polymerase chain reaction (PCR) test is performed to establish viral load and positive patients are treated with a DAA. Management is conducted either by the primary care physician, with virtual hepatology specialist consultation, or directly by a specialist, depending on liver function and degree of fibrosis, in accordance with accepted European Association for the Study of the Liver guidelines [17]. The high-risk populations outlined in the program are as follows: (1) recipients of blood products before 1992; (2) past or present injection drug users; (3) those who underwent invasive procedures in sites without proper sterilization conditions; (4) those who were born and might have underwent invasive procedures in one of the following countries, considered endemic for HCV: Armenia, Azerbaijan, Belarus, Estonia, Georgia, Kazakhstan, Kyrgyzstan, Latvia, Lithuania, Moldova, Russia, Tajikistan, Turkmenistan, Ukraine, Uzbekistan, or Romania; (5) human immunodeficiency virus (HIV) or hepatitis B virus (HBV) carriers; and (6) those who formerly tested positive for HCV but were not yet treated. In Israel, the largest of these populations is immigrants from the former Soviet-bloc states. Since 1989, over a million individuals immigrated from former Soviet Union states to Israel, amounting to as much as 10% of the population of the country [18]. The reason this population is considered at risk is the insufficient sterilization practices in these countries in the past, in particular in vaccination sterilization processes [19].

In accordance with the Ministry of Health's initiative, MHS designed and implemented an active screening program for the largest of the listed groups, immigrants from former Soviet-bloc states. Relevant members were identified for screening based on country of birth as documented by the National Insurance data. From November of 2021, HCV blood test referrals were inserted into the electronic medical records of relevant members. They were then sent text messages with information regarding HCV and the screening process and were invited for testing in the clinic. Laboratory staff were trained to explain the process to patients, and to offer the test if a relevant patient came to the lab for any other tests. If the test was not performed, relevant members continued to receive similar messages quarterly. Embedding a reminder for HCV testing [20], as well as using text messages to improve screening, has been shown to be effective in previous studies [21, 22]. The use of both of these devices, in conjunction with widespread campaigns for both physicians and patients, regular audits, stringent follow-up, and a proven effective linkage to care plan, enabled our effective intervention.

In this study, we aim to evaluate the success of the screening process as implemented by MHS before and after the intervention period in the high-risk population of former Soviet-bloc immigrants. Determining whether this process is effective in reaching the relevant population may have long-term clinical implications in the field of public health and preventive medicine. Screening for the other, much smaller at-risk groups continued via collaboration with the prison service, HCV advocacy groups, hospitals, and other settings.

## MATERIALS AND METHODS

This was a retrospective interrupted time-series (ITS) cohort study conducted by MHS, to evaluate its HCV screening program, in accordance with the national HCV eradication program. The study period was 1 June 2019 through 31 October 2024. The intervention, meaning the HCV screening program, started on 1 November 2021. The study was approved by the MHS institutional review board (MHS-0050-23).

All MHS members born in former Soviet Union states were included in the study. Participants were excluded if they already had an HCV antibody test performed in the past, or if they were under the age of 18 years.

For each participant we extracted age, sex, socioeconomic status (SES; ranked from 1 [lowest] to 10 [highest], determined based on patient addresses and categorized according to the definitions set by the Central Bureau of Statistics), country of birth, and documented coinfections with HBV and HIV (using documented diagnostic codes). Country of birth was supplied by the National Insurance Institute to ensure accuracy. Participants were classified as “preintervention” if their first HCV antibody test was between 1 June 2019 and 31 October 2021, and “postintervention” as between 1 November 2021 and 31 October 2024. For secondary outcome analysis, we further divided the postintervention group into early postintervention (1 November 2021–30 April 2022), and late postintervention groups (1 May 2022–31 October 2024). These 2 groups and their characteristics were compared in analysis, to establish differences between them.

### HCV Screening Program

The 2021 screening program was based on the care cascade put in place beginning in 2019, which included virtual hepatology consultation, decreased copayment for DAAs, complete rather than monthly DAA dispensation, dedicated teams to contact patients, and central MHS oversight. To reach target populations, the screening program added a few elements: HCV blood test referrals were put in place for the study population, and laboratory workers were trained to offer the test to eligible patients who would come to the lab for any other test.

A patient journey program was sent by test and email, informing the patients of the screening program, offering testing, and providing information about the test, hepatitis C, and treatment.

Reflex testing was implemented (automatic PCR testing for patients with positive HCV antibodies). This is reflected as the “late intervention period” in our study.

Webinars, lectures, and advertisements in Russian were carried out in areas with a high number of previous Soviet-bloc patients.

Our primary outcome was the monthly counts of anti-HCV tests performed, each for 1 patient. Secondary outcomes were HCV care cascade including anti-HCV test positivity rates, HCV PCR test positivity rates, initiation of DAAs treatment within 6 and 12 months after screening, and posttreatment HCV PCR for SVR confirmation.

Tests were conducted through the MHS central laboratory. HCV antibodies were tested with the Abbott Architect Anti-HCV kit (1 sample per cutoff). HCV viral load was tested with the Abbott Alinity m HCV AMP kit (cutoff 12 IU/mL).

Variables are reported with medians and interquartile ranges (IQRs) or counts with percentages. Sociodemographic characteristics as baselines were compared using standardized mean differences (SMDs) with values >0.1 considered meaningful imbalance. To assess effect of the program on screening results, we performed an ITS analysis [18]. Monthly anti-HCV test counts were modeled using negative binomial regression, as they showed overdispersion. Model coefficients are reported as incidence rate ratios (IRRs) with 95% confidence intervals (CIs). HCV care cascade outcomes were compared across 3 periods: (1) preintervention, (2) early postintervention, and (3) late postintervention, using χ^2^ or Kruskal–Wallis tests, as appropriate. A distinction between the early and late postintervention periods was made, as during the late postintervention period, a reflex test was performed, conducting an immediate PCR test on all positive anti-HCV results. All analyses were performed in R version 4.5.0 software. Statistical significance was set at a *P* value <.05.

## RESULTS

### Participants and Descriptive Data

A total of 335 870 MHS members were identified as immigrants from former Soviet Union states. Of these, 157 027 (46%) were tested prior to 1 June 2019, the start date of our study, and 179 658 members remained untested and met inclusion criteria. During the preintervention period, 25 613 of these individuals (14.3%) underwent HCV testing, leaving 154 046 eligible for screening at the start of the intervention period. Of this eligible pool, 122 368 (79.4%) were screened during the intervention period. Overall, by the end of the intervention period, a total of 147 980 (82.3%) of members meeting inclusion criteria had undergone screening.

Those screened at the preintervention period were younger (median age, 47 vs 57 years; SMD = 0.37) and were predominantly female (60% [n = 15 365] compared with 53% [n = 81 784] male; SMD = 0.14). The preintervention group had higher prevalences of HIV and HBV positivity, with 0.4% (n = 99) positive for HIV compared with <0.1% (n = 150), and 1.4% (n = 351) were HBV positive compared with 0.3% (n = 521). See Table 1 for full data.

**Table 1.** Characteristics of Individuals in the Preintervention and Postintervention Periods

Characteristic	Overall (n = 179 658)	Preintervention (n = 25 612)	Postintervention (n = 154 046)	SMD
Age, y, median (IQR)	55 (41–70)	47 (36–64)	57 (42–70)	−0.37
Sex, female	97 149 (54)	15 365 (60)	81 784 (53)	0.14
Birth country				0.19
Armenia	229 (0.1%)	47 (0.2%)	182 (0.1%)	…
Azerbaijan	1864 (1.0%)	287 (1.1%)	1577 (1.0%)	…
Belarus	4236 (2.4%)	696 (2.7%)	3540 (2.3%)	…
Estonia	58 (<0.1%)	10 (<0.1%)	48 (<0.1%)	…
Georgia	104 (<0.1%)	31 (0.1%)	73 (<0.1%)	…
Kazakhstan	2642 (1.5%)	382 (1.5%)	2260 (1.5%)	…
Kyrgyzstan	457 (0.3%)	77 (0.3%)	380 (0.2%)	…
Latvia	560 (0.3%)	83 (0.3%)	477 (0.3%)	…
Lithuania	438 (0.2%)	55 (0.2%)	383 (0.2%)	…
Moldova	2050 (1.1%)	340 (1.3%)	1710 (1.1%)	…
Romania	9097 (5.1%)	586 (2.3%)	8511 (5.5%)	…
Russia	22 269 (12%)	3527 (14%)	18 742 (12%)	…
Soviet Union	103 988 (58%)	14 423 (56%)	89 565 (58%)	…
Tajikistan	333 (0.2%)	42 (0.2%)	291 (0.2%)	…
Turkmenistan	347 (0.2%)	51 (0.2%)	296 (0.2%)	…
Ukraine	26 921 (15%)	4360 (17%)	22 561 (15%)	…
Uzbekistan	4065 (2.3%)	615 (2.4%)	3450 (2.2%)	…
Socioeconomic status				0.03
Low	39 078 (22%)	5662 (22%)	33 416 (22%)	…
Medium	109 349 (61%)	15 736 (61%)	93 613 (61%)	…
High	31 231 (17%)	4214 (16%)	27 017 (18%)	…
HIV carrier	249 (0.1%)	99 (0.4%)	150 (<0.1%)	0.06
HBV carrier	872 (0.5%)	351 (1.4%)	521 (0.3%)	0.11

Data are presented as No. (%) unless otherwise indicated.

Abbreviations: HBV, hepatitis B virus; HIV, human immunodeficiency virus; IQR, interquartile range; SMD, standardized mean difference.

### Main Results

**Figure 1.**
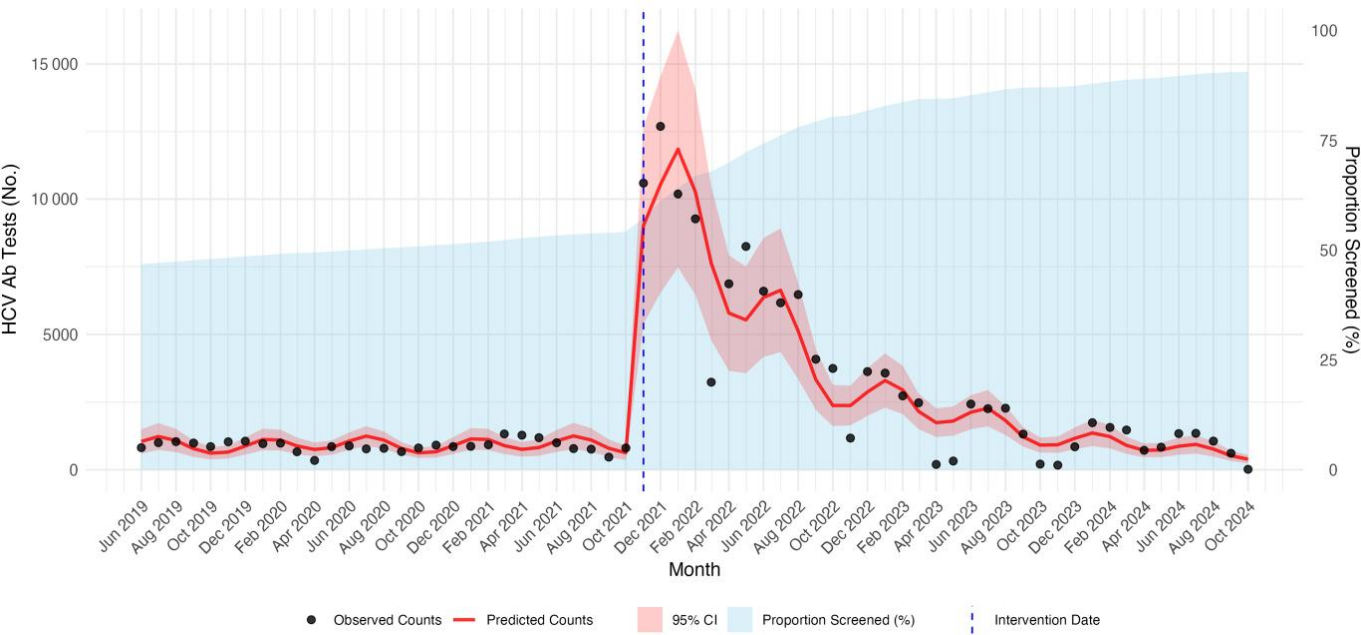
Monthly hepatitis C virus antibody (HCV Ab) screening tests in the target population before and after intervention, with interrupted time-series model predictions. Black points represent observed monthly anti-HCV Ab test counts. The dashed vertical blue line marks the intervention date. The red line represents predicted monthly counts from the interrupted time-series negative binomial regression model, and the red shaded area indicates the 95% confidence interval (CI) of the model predictions. The light blue shaded area (right y-axis) shows the cumulative proportion of the target population screened over time (%), beginning at 50% representing the Maccabi Health Services previous Soviet-bloc population that had HCV testing in the past for any reason.

From the start of the intervention to the end of the study period, 122 368 MHS members were screened for HCV, raising cumulative coverage to 147 980 (82.3% of the at-risk population at the start of the preintervention period). Monthly screening volume surged immediately after the launch of the intervention, peaking in January 2022, with >10 000 participants screened. Monthly screening rates later plateaued at 1000–2000 tests per month (Figure 1).

In ITS analysis of monthly anti-HCV antibody testing counts, the intervention was associated with a marked immediate increase in testing volume (postintervention level change IRR, 67.2 [95% CI, 28.3–162]; *P* < .001), followed by a significant declining postintervention trend relative to the preintervention slope (postintervention × time IRR, 0.95 per month [95% CI, .92–.98]; *P* < .001). The baseline preintervention monthly trend was not statistically significant (time IRR, 1.01 [95% CI, .98–1.03]; *P* = .6). Full ITS model coefficients are provided in Supplementary Table 1.

Out of 147 980 screened individuals, 1964 (1.33%) were HCV positive according to the serology test. Rates of positive results fluctuated significantly over time. Before the intervention, 2.6% of participants were HCV positive; in the early postintervention period this rate decreased to 1%, and 1.1% in the late postintervention period (*P* < .001). While positive serology results have to be confirmed with a PCR test [15], 1831 (93.2%) of the positively tested individuals did a confirmatory PCR. The overall rate of positive PCR results was 51.7% and did not significantly change over the study period (*P* = .075).

**Figure 2.**
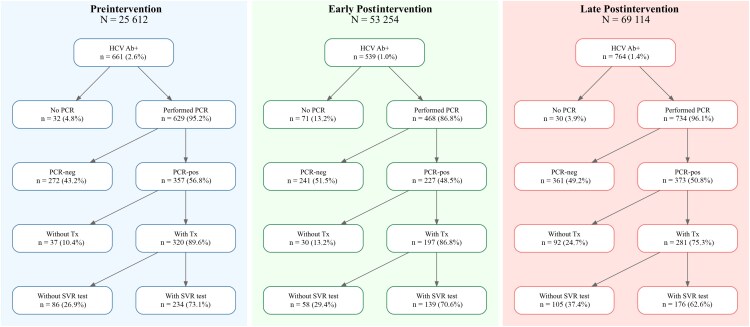
HCV care cascade outcomes across study periods. The cascade is shown for the preintervention period and for the postintervention period, which was subdivided into the period before implementation of HCV PCR reflex testing (early postintervention) and the period following implementation of reflex testing (late postintervention). Panels display the number and proportion of individuals at each step of the cascade, including HCV Ab positivity, PCR testing, PCR result, treatment within 12 months among those with positive PCR, and SVR testing among those treated. Percentages at each step are conditional on the immediately preceding stage. Abbreviations: HCV Ab+, hepatitis C virus antibody positive; PCR, polymerase chain reaction; SVR, sustained virologic response; Tx, treatment.

Of 947 participants who tested positive for HCV, 798 (84.3%) started treatment. The treatment rate was 92% (n = 320) before the intervention, 87% (n = 197) in the early postintervention period, and 75% (n = 281) in the late postintervention period (*P* < .001). Treatment started within 6 months for 79% (n = 630) and within 12 months for 90% (n = 716), without significant differences between study periods (*P* = .11 for the 6-month period and *P* = .2 for the 12-month period). Posttreatment confirmatory PCR to ensure cure (SVR) was performed in 69% (n = 549) of cases overall, with the highest rate of confirmatory PCR tests in the preintervention period and the lowest rate in the late postintervention period (73% [n = 234], 71% [n = 139], and 63% [n = 176], respectively; *P* = .018). Median time from treatment completion to confirmatory PCR was 106 days (IQR, 86–145 days) and was similar between groups. See Figure 2 and Supplementary Table 2 for full data.

## DISCUSSION

In this retrospective ITS cohort study, we evaluated the implementation and outcomes of MHS’ HCV screening program for former Soviet-bloc immigrants, launched in November 2021. We evaluated the program and its performance for 3 years, until the end of October 2024. Outcomes were compared to the period prior to launch of the program when HCV testing was primarily indicated in the context of a clinical investigation. The strategy implemented here integrated multiple system-level components: automated test referrals that eliminated the need for clinician-initiated orders, culturally tailored educational messaging, and repeated direct-to-patient digital outreach, within a single coordinated framework. This comprehensive design reduced structural and behavioral barriers simultaneously, enabling screening to occur without appointments or physician visits, a feature not commonly evaluated in national programs

Our findings are divided into 2 primary categories: the characteristics of the tested population before and after the intervention, and the evaluation of the implemented program as a preventive medicine strategy.

### Population Characteristics

Interesting features of the preintervention sample are younger age and female dominance. This may be explained by a decision taken in MHS in 2021, to add HCV screening as part of the routine pregnancy and prepregnancy panel, based on findings and recommendations for at-risk groups [23, 24]. This is indicated due to the potential adverse effects of HCV on pregnancy and neonatal outcomes, including low birth weight, small for gestational age, need for ventilation, and neonatal intensive care [25, 26]. The higher proportion of females in the preintervention stage might reflect physicians ordering the test for this demographic before formal screening, whereas postintervention the percentage becomes closer to the national sex ratio of 50.4% female to 49.6% male [27]. This may also account for the younger age group found in the preintervention group, as more tests were conducted in the fertile age group.

The higher rates of HIV and HBV in the preintervention group is not surprising, as the initial workup was most likely driven by clinical suspicion, with hepatitis viruses and HIV being part of the infectious panel. Furthermore, as these 3 viruses have common modes of transmission, it is not unlikely to find coinfection [28].

Another interesting finding is that SES was not found to affect HCV screening before the intervention, with similar distribution between preintervention and postintervention groups. Risk factors for HCV infection can be associated with socioeconomic features and some evidence suggests that SES is an independent risk factor [29, 30], so it might have been expected that the preintervention group would have different characteristics. Since testing before the intervention is based on clinical decisions, the reasons for testing can shed more light on the specific composition of at-risk groups in Israel. While healthcare in Israel is universal, it is possible that specific circumstances such as stigma or poor health literacy might decrease the rate of the very low SES population being tested in the HMO, and instead opting for free clinics, testing within the prison services, or not utilizing nonurgent healthcare services [31, 32]. This potential omission of a certain portion of at-risk patients can confound the true characteristics of the examined population; this can likewise be established by the clinical considerations for the test in the preintervention group.

### Screening Program Evaluation

Our study findings highlight the success of our targeted screening and eradication program. By the end of the study period, 3 years after the launch of the program, 82.3% (n = 147 980) of the study population was screened, meaning that >90% of the target population (all immigrants from former Soviet-bloc states in MHS) was tested. By itself, this finding indicates that the intervention had achieved its main purpose: screening the at-risk population. We attribute this success to several features known to increase adherence to screening practices. First, patients were informed about the disease and its potential risks through text messaging, lectures, publications, etc [33]. Second, the test was done easily at the local clinic, without the need to request a referral, significantly reducing associated “hassle” [34]. Third, reminders were sent, to encourage patients who did not act immediately upon receiving the invitation for screening [35]. The program continued to include regular follow-ups and analyses of implementation and treatment by dedicated MHS teams at the district and national levels. Auditing was further conducted by the Ministry of Health and data were periodically transferred. Elements of this decentralized, integrated approach have also been used successfully in countries such as Georgia, Rwanda, and Nigeria [36], with screening targets reached up to 80%. We attribute our success at screening >90% of our target population in a relatively short period of time to the unique combination of steps put in place integrating patient, provider, and physician needs for a successful screening program.

The rate of positive tests was significantly lower among the postintervention group compared to those tested preintervention. As noted, preintervention testing was often diagnostic rather than preventive, meaning clinical suspicion of physicians led them to test specifically for HCV. Understandably, those prompted to be tested due to specific exposures or symptoms are at greater risk for a positive result. Regarding screening practices in general, we expect to find far fewer positive cases than in the population tested due to clinical suspicion [37]. Once testing for a condition is clinically indicated, the condition might already be symptomatic and advanced. Ideally, screening practices detect conditions when “silent” and not clinically indicated. The rate of positive HCV tests (about 1%) postintervention may also include those tested due to clinical indications if they were also included in the criteria of the screening population. However, the postintervention group was >6 times the size of the preintervention population. This proportion, compared with the rate of positive tests before and after the intervention (2.6% and 1%–1.1%, respectively) indicates that it is likely many, if not most, of the positive HCV tests were not clinically indicated (ie, screening tests).

The peak of screening occurred soon after program launch, with >10 000 tests performed. This initial effect is significant and represents the first introduction of the information to the target population. A challenging population, however, is those who did not act upon the initial invitation. Nevertheless, even after the initial surge, we can see a steady stream of screened patients, with peaks every 3 months, correlating to the times reminders were sent. Trends of adherence after the initial surge should be analyzed and perhaps may reveal more about potential motivations for harder-to-reach groups.

Worldwide, only about 20% of diagnosed HCV patients receive treatment as of 2024 [1]. In our study, the overwhelming majority of patients who tested positive in the subsequent PCR test were treated, via an ongoing care process in MHS. However, we do see a clear difference between the preintervention and postintervention groups in overall rate of treatment. Moreso, even in the postintervention group, the early postintervention subgroup had significantly higher treatment rates (87%) compared to the late postintervention subgroup (75%). However, results are comparable between the groups when looking at time to treatment among patients treated. The proportion of patients in the late postintervention group to receive treatment within 6 months (82%) or 12 months (92%) is higher than in the other groups.

The overall lower number of treated patients in the early (87%) and late (75%) postintervention groups, as compared to the preintervention group (92%), may reflect the time needed to convince patients who have no symptoms of the need for treatment. Presence of symptoms and detected liver injury are known to increase adherence to HCV treatment [38].

Lower adherence to treatment in the late postintervention subgroup might also be correlated with their relatively delayed adherence to screening; meaning as they did not urgently seek to act upon the screening invitation, they might not urgently seek to treat the condition. Whether they choose to be treated later can shed more light on their “cumulative” adherence. Another possibility explaining the lower treatment rates in the late intervention group is the shorter time in which our teams had to follow up and encourage treatment uptake. Understanding the trends and potential barriers to treatment in this population is important and should be prioritized in future studies.

The eradication of HCV in Israel and globally is a multistep process that requires screening, early detection, and treatment. We report that actively approaching patients, removing bureaucratic barriers, and regularly reminding them about screening are highly effective measures in the screening process. These can lead to treatment for most HCV carriers, reducing the incidence of this potentially severe disease. The study highlights how a tailored structured process is successful in screening programs and can be implemented in additional screening programs nationally and internationally in the future.

### Limitations and Strengths

The study is based on data available to MHS and may possibly have excluded patients who performed the test in other settings. SVR data are limited, and further tests may have been conducted outside the study timeline. It is also possible other tests and treatments were done in external medical settings, though it is unlikely due to costs and services available privately in the country. The rationale for testing was not examined per patient, and preintervention may have included patients who underwent screening, as postintervention may have included clinical suspicion as well. Although MHS is very well represented in the country, it has a smaller representation in the periphery, potentially causing a bias in patient population. In addition, no data were collected regarding patient drug use. Furthermore, the question of SES and other demographic factors as possible variables to screening response was not examined.

However, this study also has notable strengths. First, it was done on a large population, representing the at-risk population locally. Additionally, we were able to establish that screening and actively approaching individuals results in reaching a distinct group, which differs from those tested for HCV before the implementation of the program. Third, we evaluated not only the screening itself, but also adherence to treatment and confirmatory tests. Finally, access to the medical record of all patients allowed for a more comprehensive understanding of the population’s characteristics. Importantly, the magnitude of the effect, moving from 14% to over 80% screening coverage, demonstrates what can be achieved when reminders are paired with the removal of bureaucratic steps and with patient-centered communication. These findings also have implications for global generalizability. Although local health system infrastructure may vary, the core elements of the intervention, such as automated eligibility identification, barrier-free test ordering, and direct engagement using widely accessible technologies such as text messaging, are scalable and could be adapted by other countries seeking to accelerate HCV elimination. Based on these results, direct-to-patient text outreach appears to be a highly efficient and acceptable method for improving screening uptake and may be recommended as part of national strategies, provided that similar digital capabilities and population-specific adaptations can be implemented

## CONCLUSIONS

In this interrupted time-series cohort study, we evaluated the implementation of an HCV screening program. Before the intervention, only 14.2% of the relevant population was tested for HCV; within 3 years the intervention increased this portion to >82%. The majority of those who tested positive for HCV were duly treated. We can report that actively reaching out to patients, removing bureaucratic barriers, and sending reminders routinely are viable strategies for HCV screening.

## Supplementary Material

ofag278_Supplementary_Data

## Supplementary Data

Supplementary materials are available at *Open Forum Infectious Diseases* online. Consisting of data provided by the authors to benefit the reader, the posted materials are not copyedited and are the sole responsibility of the authors, so questions or comments should be addressed to the corresponding author.
