# Supplementary material for: Implementation of a Hepatitis C Screening Program for At-Risk Former Soviet-Bloc Immigrants in a Large Health Maintenance Organization
Source: Open Forum Infect Dis. 2026 May 6;13(5):ofag278. doi: 10.1093/ofid/ofag278 (PMC13197195; doi:10.1093/ofid/ofag278)
Supplement: ofag278_Supplementary_Data [file ofag278_supplementary_data.docx]

| **Characteristic** | **IRR** | **95% CI** | **p-value** |
| --- | --- | --- | --- |
| post_intervention | 67.2 | 28.3, 162 | <0.001 |
| time | 1.01 | 0.98, 1.03 | 0.6 |
| fourier |  |  |  |
| fourierS1-12 | 0.99 | 0.83, 1.19 | >0.9 |
| fourierC1-12 | 1.11 | 0.93, 1.34 | 0.2 |
| fourierS2-12 | 1.22 | 1.02, 1.46 | 0.025 |
| fourierC2-12 | 0.82 | 0.68, 0.98 | 0.025 |
| post_intervention time | 0.95 | 0.92, 0.98 | <0.001 |
| Abbreviations: CI = Confidence Interval, IRR = Incidence Rate Ratio | | | |

Supplementary Table 1- Interrupted time sequence model coefficients

| **Intervention outcome** | **Overall** N = 147,980*^1^* | **Pre Intervention 11-2021** N = 25,612*^1^* | **Early Post-Intervention** N = 53,254*^1^* | **Late Post -Intervention** N = 69,114*^1^* | **p-value***^2^* |
| --- | --- | --- | --- | --- | --- |
| Positive HCV Ab tests of those performed | 1,964 (1.3%) | 661 (2.6%) | 539 (1.0%) | 764 (1.1%) | <0.001 |
| Performed HCV PCR test of those with positive HCV Ab | 1,831 (93%) | 629 (95%) | 468 (87%) | 734 (96%) | <0.001 |
| Positive HCV PCR tests of those performed | 947 (52%) | 347 (55%) | 227 (49%) | 373 (51%) | 0.075 |
| Positive PCR in each group who received treatment | 798 (84%) | 320 (92%) | 197 (87%) | 281 (75%) | <0.001 |
| Positive PCR who received treatment within 6 months of total treated in each group | 630 (79%) | 241 (75%) | 159 (81%) | 230 (82%) | 0.11 |
| Positive PCR who received treatment within 12 months of total treated in each group | 716 (90%) | 283 (88%) | 174 (88%) | 259 (92%) | 0.2 |
| Treated patients who repeated the test (SVR) | 549 (69%) | 234 (73%) | 139 (71%) | 176 (63%) | 0.018 |
| Time from treatment to repeated test (days) | 106 (86, 145) | 100 (84, 135) | 110 (83, 148) | 110 (90, 149) | 0.073 |
|  |  |  |  |  |  |
|  |  |  |  |  |  |

*Supplementary Table 2:*  *HCV care cascade outcomes across study periods. Post-intervention results were divided into pre-HCV PCR reflex testing (early post-intervention) and following reflex testing (late post-intervention).*

*^1^n (%); Median (Q1, Q3)*

*^2^Pearson's Chi-squared test; Kruskal-Wallis rank sum test*
